# Acute Intermittent Porphyria Associated with Respiratory Failure: A Multidisciplinary Approach

**DOI:** 10.1155/2011/283690

**Published:** 2011-05-11

**Authors:** Mayra Gonçalves Menegueti, Alkmim-Teixeira Gil Cezar, Karin Aparecida Casarini, Kátia Simone Muniz Cordeiro, Anibal Basile-Filho, Olindo Assis Martins-Filho, Maria Auxiliadora-Martins

**Affiliations:** ^1^Divisão de Terapia Intensiva, Departamento de Cirurgia e Anatomia, Hospital das Clínicas, Faculdade de Medicina de Ribeirão Preto, Universidade de São Paulo, 14049-900 Ribeirão Preto, SP, Brazil; ^2^Laboratório de Biomarcadores de Diagnóstico e Monitoração, Instituto René Rachou, Fundação Oswaldo Cruz, Belo Horizonte, MG, Brazil

## Abstract

Despite being challenging, delivery of effective nursing care to patients with acute intermittent porphyria is a matter of utmost importance. In this paper, the diversity of symptoms and the difficult diagnosis of this condition are emphasized, and details concerning the treatment of this disorder in the intensive care unit are presented. We believe that acute intermittent porphyria should be borne in mind during performance of differential diagnosis of neurological, psychiatric, and gastroenterological disorders on patients whose routine investigation tests are normal, especially when precipitating factors exist. Intensive care measures and a multidisciplinary team approach are essential.

## 1. Introduction

Acute intermittent porphyria (AIP) is an autosomal dominant disorder caused by deficiency of porphobilinogen deaminase (PBGD), which is involved in the hepatic heme biosynthesis [1]. Decreased PBGD activity leads to elevated urine levels of porphyrin precursors, namely, delta-aminolevulinic acid (ALA) and porphobilinogen (PBG), which can be evidenced by the Watson-Schwartz test [2]. There are three autosomal dominant acute porphyrias: AIP, variegate porphyria, and hereditary coproporphyria. Symptomatic AIP prevalence is 2 to 3 cases per 100,000 persons per year [3].

Porphyria attacks can be triggered by environmental factors, reduced calorie intake [4], medications (barbiturate, calcium channel blockers, antibiotics, antifungals, and hormones) [5], large alcohol intake, nicotine abuse, infection, surgery, or psychological disorder. Endocrine factors and steroid hormones may also be the cause of attacks. 

The most frequent clinical manifestation of acute attacks include vomiting, hypertension and tachycardia [6], acute abdominal pain (incidence estimated at 85%–95%) [7], and peripheral neuropathy with muscle weakness (42%–68%). Respiratory failure is less common and less known (9%–20%). The exact mechanism through which these signs and symptoms occur remains unknown [8]. 

Treatment of this condition consists in eliminating the precipitating factors, avoiding drugs that may trigger a crisis, and treating possible infections. High-carbohydrate diet, glucose infusion, or hematin infusion can also be prescribed.

Despite being challenging, effective nursing care delivery to patients with acute intermittent porphyria is a matter of utmost importance. In the present paper, the diversity of symptoms and the difficult diagnosis of this condition are emphasized, and details concerning the treatment of this disorder in the intensive care unit using a multidisciplinary approach are presented.

## 2. Cases Description

### 2.1. Patient 1

A white female patient aged 24 years and with a family history of AIP was admitted to the intensive care unit (ICU) complaining of abdominal pain that had been ongoing for the five previous months. She had been initially treated with proton pump inhibitors and antispasmodic medication in her hometown. Over the previous three months she had evolved with diarrhea, vomiting, and progressive flaccid tetraparesis. Her condition had worsened over the previous 15 days, with involvement of respiratory muscles and need for mechanical ventilation. Physical examination revealed a conscious patient that responded with movements of the head only. She presented with a Glasgow coma scale of 8, flaccid tetraplegia, facial hemiparesis, and hypoglossal paralysis, but was hemodynamically stable (with BP = 125/70 mmHg and heart rate of 110 bpm). Chest auscultation showed diffuse wheezes; heart and abdomen auscultation was normal. The APACHE II score [9] was 20.

Blood lead level gave no evidence of heavy metal poisoning. Lumbar puncture with cerebrospinal fluid analysis showed absence of Guillain-Barre syndrome. On the basis of the patient's clinical and familial history, the presence of AIP was investigated, which included determination of delta-aminolevulinic acid (ALA) (39.6 mg/g creatinine—normal value: 4.5 mg/g creatinine) and porphobilinogen (PBG) (17.75 mg/day—normal value: 3.5 mg/day) upon admission to the ICU. Electromyography results demonstrated the presence of severe sensory motor axonal polyneuropathy.

On the basis of the clinical findings and complementary examinations, a high-carbohydrate diet (400 to 450 g/day) was started, as recommended for AIP cases. Treatment with hematin was only initiated later, due to the difficulty in obtaining the drug, and it was maintained for 14 days.

During her stay in the ICU, the patient progressed with symptoms of urinary tract infection and pneumonia associated with mechanical ventilation. The patient was dismissed from the ICU after a 107-day stay and discharged from hospital 79 days later. On her last visit to the outpatient clinic, she was capable of ambulating short distances. She was able to carry out personal hygiene and feed herself with assistance.

### 2.2. Patient 2

A 47-year-old male patient with a history of alcoholism was admitted to the ICU with initial symptoms of diffuse strong abdominal pain that had started 15 days previously. Approximately 5 days prior to admission, the patient had presented with trembling and visual hallucinations, as well as pain and weakness in the inferior limbs. He was initially treated for alcohol abstinence in his hometown. There was a sharp increase in muscle weakness, which demanded orotracheal intubation, sedation, and invasive mechanical ventilation three days after ICU admission. Physical examination revealed a conscious patient with periods of spatial and temporal disorientation. His Glasgow coma scale was 14, and the respiratory rate was 28 bpm, aided by accessory muscles, BP = 188/96 mmHg, heart rate = 138 bpm. Respiratory auscultation demonstrated diffuse wheezes; heart and abdomen auscultation was normal. The APACHE II score was 17.

Blood lead level gave no evidence of heavy metal poisoning. Lumbar puncture with cerebrospinal fluid analysis showed absence of Guillain-Barre syndrome. PCR tests for enterovirus, Epstein-Barr virus, cytomegalovirus, and herpes simplex viruses were negative. Serology for HIV was also negative. The computed tomography scan was normal. The presence of AIP was investigated, which included dosage of delta-aminolevulinic acid (ALA) levels on the second and sixth day after ICU admission (17.8 and 28.5 mg/day, respectively—normal range: 1.3 to 7 mg/day) and determination of porphobilinogen (PBG) levels on the second day of ICU stay (2.52 mg/day normal range: 0.0 to 2 mg/day). The Erlich test was positive, and hyponatremia persisted during the ICU stay. Electromyography results revealed the presence of sensory motor axonal polyneuropathy and asymmetric neuropathy with signs of acute and chronic axonal loss.

On the basis of the clinical findings and complementary examinations, a high-carbohydrate diet (400 to 450 g/day) was started, as recommended for AIP cases. Treatment with hematin was only initiated after 20 days of ICU admission, due to the difficulty in obtaining the drug, and it was maintained for 9 days.

The patient was discharged from the ICU after a 54-day stay and was discharged from hospital 20 days later. He was breathing spontaneously, but presented motor deficit and difficulty in ambulating.

### 2.3. Patient 3

A male patient aged 31 years was admitted to the ICU with a one-month history of urine retention and constipation accompanied by abdominal pain and famiy history of similar cases, but with no confirmed diagnosis. The patient had spent twenty days at an ICU in his hometown, where he tested positive for the Erlich test. Determination of delta-aminolevulinic acid (ALA) (32 mg/day—normal range: 1.3 to 7 mg/day) and porphobilinogen (PBG) (2.4 mg/day—normal range: 0.0 to 2 mg/day) levels was also performed at his hometown, where AIP was suspected. After a few days, the patient presented with weakening of the inferior limbs. After another day, he presented tetraplegia without sensitivity loss, hoarseness, dysphagia, and diplopia. The patient underwent tracheostomy and mechanical ventilation due to globally reduced muscle strength.

Physical examination at the ICU revealed that the patient was awake and conscious, and he responded to verbal stimuli. His Glasgow coma scale was 10, BP = 140/80 mmHg, and HB = 98 bpm. Heart, respiratory, and abdomen auscultation was normal. The APACHE II score was 10.

Electromyography revealed a normal axonal pattern with associated denervation of severe intensity. Lead dosage was normal.

On the basis of the clinical findings and complementary examinations, a high-carbohydrate diet (400 to 450 g/day) was started, as recommended for AIP cases. Treatment with hematin was only initiated after 20 days of ICU admission, due to the difficulty in obtaining the drug, and it was maintained for 8 days.

The patient was discharged from the ICU after a 17-day stay, although he was under mechanical ventilation with Bi-level Positive Airway Pressure (BIPAP), and he was discharged from hospital 33 days later.

On his last visit to the outpatient clinic, he was still tracheostomized. He was being assisted by a nurse, a physiotherapist, and a phonoaudiologist, was being fed orally, and presented significant clinical improvement, being able to ambulate without assistance.

## 3. Discussion

Under normal conditions, enzyme deficiency alone is not sufficient to trigger a porphyria attack. Generally, the presence of other precipitating factors is necessary to induce appearance of symptoms. Around 80% of the people with deficiency of enzymatic activity never present any symptoms (latent AIP), whereas others experience only occasional, slight symptoms [8].

The clinical history of AIP varies widely and shares many symptoms and signs common to other disorders, as observed in the three cases reported here. Therefore, diagnosis is based on laboratory criteria such as elevated excretion of the porphyrin precursors ALA and PBG in the urine [10]. Accumulation of these precursors in the urine may change the urine color after sun exposure, from yellowish to dark red or brown, and occasionally purple. From a laboratory viewpoint, a largely elevated PBG level in 24 h urine is a diagnosis of acute porphyria. 

Of the three reported cases, two presented two signs and symptoms of the disease that is, abdominal pain and high blood pressure [11]. The three cases involved respiratory failure that demanded mechanical ventilation. In case 1, the triggering factor was not identified; alcohol intake was the probable cause of the porphyria attack in case 2; significant stress factors within the family setting were the reason for the crisis in case 3. Attention must be given to abdominal pain symptoms in unusual attacks in critical patients, and AIP should be suspected. When analyzed in isolation, the patients' symptoms may mimic several disorders of the digestive system, so a high level of suspicion is mandatory. In this sense, diagnosing porphyria is a difficult task that on many occasions is accomplished only after several attacks of the disease. In an early phase, AIP may be hypothesized and early diagnosed when there is a family history of the condition, which was the case of patient 1. 

 Treatment of AIP consists in the management of the symptoms with safe medications, discontinued use of porphyrinogenic drugs, elevated carbohydrate supply, and hematin infusion. The latter drug inhibits the action of the first enzyme involved in the heme synthetic pathway, thereby blocking porphyrin production and accumulation. This therapy was successfully applied in the three cases reported herein. 

Because AIP diagnosis requires several complementary analyses and the therapeutic protocols incur extremely high costs, such services are only available in major hospitals of large cities in Brazil. The access to early diagnosis is difficult for most individuals living outside the metropolitan areas. Early AIP diagnosis and prompt prescription of specific therapies along with implementation of a family assistance program are extremely important for the successful management of this condition [12]. 

Because AIP is little known to the lay population and since its repercussions are potentially fatal, an interdisciplinary approach that ensures adequate medical, nursing, and psychotherapeutic support is crucial during care delivery to AIP patients. In the present cases, the multiprofessional team working at our ICU designed collaborative strategies for patient treatment. The extremely rare acute occurrence of this disease implies that most of the patients and relatives do not have any knowledge of this pathology or its evolution, which results in fear and insecurity. Therefore, the multiprofessional staff must be prepared to give information about this illness and its progression. 

Our experience showed that it is useful to initially inform relatives of the reasons for patient transfer to the ICU and explain their symptoms, especially those related to dependence and discomfort, such as pain and respiratory failure. Psychological support to the relatives is also an important therapeutic tool, since it promotes closer and more positive interactions among relatives, the patient, and the multiprofessional team, thus favoring formation of therapeutic alliances. 

One of the aims of the nursing team during the management of AIP patients is to design an action plan that will detect problems and establish interventions that will improve patient conditions (nursing assistance systematization). AIP may result in patient inability to maintain spontaneous breathing, which will demand constant monitoring of the vital signs and breathing pattern. 

Moreover, many conditions, including AIP, lead to impaired physical mobility, not to mention that the patient may be at risk of developing pressure ulcers. Therefore, it is essential that every two hours the patient is assisted with changing positions, in order to prevent decubitus. Patient mobilization could also be fostered by helping the individuals to leave the bed or even by taking them on wheelchair rides outside the ICU environment. These procedures can be organized with the collaboration of the entire multiprofessional team. The team must also be ready to detect signs of fear and anxiety that are commonly related to the sudden appearance of symptoms and the loss of functional abilities. 

Although most AIP patients rarely experience an attack, other diseases such as chronic renal failure, systemic arterial hypertension, and hepatocarcinoma may occur during porphyria progression [8]. Prevention of new attacks is as important as the early AIP diagnosis and treatment, thereby avoiding complications. Therefore, medical followup associated with such simple measures as attention to the diet avoidance of porphyrinogenic drugs, alcohol, and tobacco and stress relief are mandatory. It is also important that all the AIP patients' relatives are evaluated, even if they are asymptomatic, in order to verify whether the genetic defect [13, 14] is present and, if so, to start prevention. Finally, a multidisciplinary evaluation involving the neurology staff is important, so that preventive measures can be taken early, thereby avoiding patient hospitalization in the ICU. Also, patients should be discharged from hospital as soon as possible, in order to prevent complications related to long ICU stay, such as hospital infections. Patient followup at home via home care should be encouraged.

## 4. Conclusion

We believe that intensivists should suspect AIP in the differential diagnosis of neurological, psychiatric, and gastroenterologic disorders in the case of patients whose routine tests are normal, especially when precipitating factors exist. ICU support measures along with the work of a multiprofessional staff are essential.
